# Genetic variants in *MIR17HG* affect the susceptibility and prognosis of glioma in a Chinese Han population

**DOI:** 10.1186/s12885-020-07417-9

**Published:** 2020-10-09

**Authors:** Jigao Feng, Yibin Ouyang, Dedong Xu, Qinglong He, Dayuan Liu, Xudong Fan, Pengxiang Xu, Yehe Mo

**Affiliations:** Department of Neurosurgery, the Second Affiliated Hospital of Hainan Medical University, #48 Baishuitang Road, Haikou, 570311 Hainan China

**Keywords:** Glioma, *MIR17HG*, Genetic variants, Susceptibility, Prognosis

## Abstract

**Background:**

lncRNA MIR17HG was upregulated in glioma, and participated in promoting proliferation, migration and invasion of glioma. However, the role of *MIR17HG* polymorphisms in the occurrence and prognosis of glioma is still unclear.

**Methods:**

In the study, 592 glioma patients and 502 control subjects were recruited. Agena MassARRAY platform was used to detect the genotype of *MIR17HG* polymorphisms. Logistic regression analysis was used to evaluate the relationship between *MIR17HG* single nucleotide polymorphisms (SNPs) and glioma risk by odds ratio (OR) and 95% confidence intervals (CIs). Kaplan–Meier curves, Cox hazards models were performed for assessing the role of these SNPs in glioma prognosis by hazard ratios (HR) and 95% CIs.

**Results:**

We found that rs7318578 (OR = 2.25, *p* = 3.18 × 10^− 5^) was significantly associated with glioma susceptibility in the overall participants. In the subgroup with age <  40 years, rs17735387 (OR = 1.53, *p* = 9.05 × 10^− 3^) and rs7336610 (OR = 1.35, *p* = 0.016) were related to the higher glioma susceptibility. More importantly, rs17735387 (HR = 0.82, log-rank *p* = 0.026) were associated with the longer survival of glioma patients. The GA genotype of rs17735387 had a better overall survival (HR = 0.75, log-rank *p* = 0.013) and progression free survival (HR = 0.73, log-rank *p* = 0.032) in patients with I-II glioma. We also found that rs72640334 was related to the poor prognosis (HR = 1.49, Log-rank *p* = 0.035) in female patients. In the subgroup of patients with age ≥ 40 years, rs17735387 was associated with a better prognosis (HR = 0.036, Log-rank *p* = 0.002).

**Conclusion:**

Our study firstly reported that *MIR17HG* rs7318578 was a risk factor for glioma susceptibility and rs17735387 was associated with the longer survival of glioma among Chinese Han population, which might help to enhance the understanding of *MIR17HG* gene in gliomagenesis. In subsequent studies, we will continue to collect samples and follow up to further validate our findings and further explore the function of these MIR17HG SNPs in glioma in a larger sample size.

## Background

Glioma is the most frequent neoplasms originated from neuroglial stem or progenitor cells, accounting for 80% of primary malignant brain cancers with approximately 101,600 individuals diagnosed in China each year [1, 2]. Despite the efforts of diagnosis and therapeutics, the prognosis of glioma is still depressing. Until now, the aetiology of glioma remains unclear. However, environmental and occupational exposures have been identified to be associated with the occurrence and development of glioma, especially high-dosage ionizing radiation [3]. In addition, genetic factors are also given a pivotal contribution to the occurrence and prognosis of glioma [4–6]. Several association studies have revealed that single nucleotide polymorphisms (SNPs) were associated with glioma risk and survival [7–9].

*MIR17HG* gene, located on chromosome 13q31.3, is the host gene of the microRNA 17–92 cluster. Functional studies have confirmed that the *MIR17HG* gene might be related to cell survival, proliferation, differentiation, and angiogenesis [10]. LncRNA MIR17HG, also as a long noncoding RNA which regulating the expression of miRNA, played a carcinogenic effect in various cancers including rectal cancer, gastric cancer, and lung cancer [11–13]. A recent research showed that lncRNA MIR17HG was overexpressed in glioma, and lncRNA MIR17HG knockdown inhibited the proliferation, migration and invasion of glioma, suggesting that lncRNA MIR17HG might facilitate the malignant progress of glioma [14]. Recently, increasing evidences indicated that genetic polymorphisms of *MIR17HG* were associated with the occurrence of multiple tumors, such as lymphoma, colorectal cancer, breast cancer [15–17]. However, the role of *MIR17HG* variants in glioma occurrence and prognosis is still unclear.

Here, we analyzed the association of selected *MIR17HG* SNPs and glioma susceptibility among the Chinese Han population, and examined the possible role of these polymorphisms in different glioma subgroups stratified by age, gender and grade. We also evaluated the influence of *MIR17HG* genetic variants on the survival of glioma patients.

## Methods

### Subjects

This study recruited 592 glioma patients and 502 control subjects. All participants were genetically unrelated Chinese Han population. Glioma patients who diagnosed and confirmed by histopathology were enrolled from the department of Neurosurgery at Tangdu Hospital from February 2014 to March 2018. Patients with history of cancer and other systemic or complex diseases were excluded. Age- and gender-matched healthy controls were recruited from the physical examination center of the hospital. The controls were free from any cancer and any disease related to brain and central nervous system. Standardized questionnaires and medical records were used to collect demographic and clinical information. The follow-up information was obtained by telephone and return visit every 3 months; and the survival time, progress and outcome were recorded. After, approximately 5 mL blood samples were collected for further analysis. Our study was approved by the Ethics Committee of the Second Affiliated Hospital of Hainan Medical University and was in the Declaration of Helsinki. Written informed consent was obtained from each participant.

### Genotyping

Genomic DNA was purified by a commercially available GoldMag DNA Purification Kit (GoldMag Co. Ltd., Xi′an City, China). NanoDrop 2000 (Thermo Scientifc, Waltham, MA, USA) was used to check DNA quality. Five *MIR17HG* SNPs (rs17735387, rs72640334, rs7318578, rs7336610, and rs75267932) were identified based on the NCBI dbSNP database, the 1000 Genomes Project data with minor allele frequencies (MAFs) > 5% in Chinese Han Beijing (CHB) population and Haploview software with a pairwise linkage disequilibrium (r^2^ > 0.80). *MIR17HG* polymorphisms were genotyped using Agena MassARRAY platform (Agena, San Diego, CA, U.S.A.) as previously described [18]. The primers sequences were presented in Supplementary Table 1. Genotyping was in a blinded manner, and the call rate was ≥0.99. For quality control, 10% of blind and random samples were repeated genotyping, and the result was 100% reproducibility.

### Data analysis

Statistical analysis were performed using SPSS version 18.0 (SPSS Inc., Chicago, IL, USA) and PLINK 2.1.7 package. The Chi square test or Student’s t-test was carried out to compare the differences in age and gender distributions between patients and controls, as appropriate. Hardy–Weinberg equilibrium (HWE) was performed for the controls using goodness-of-fit χ^2^ test. Logistic regression analysis was used to analyze the genetic effects of *MIR17HG* SNPs on the risk of glioma by calculating odds ratio (OR) and 95% confidence intervals (CIs) adjusted for age and sex. Multiple testing correction was performed by the false discovery (FDR). The overall survival (OS) and progression-free survival (PFS) of glioma patients were plotted by Kaplan–Meier survival curves. Univariate and multivariate Cox proportional hazards models were performed to assess the role of *MIR17HG* polymorphisms in the prognosis of glioma by calculating hazard ratio (HR) and 95% CIs. A two-tailed *p* value of < 0.05 was statistically significant.

## Results

### Participants’ features

The characteristics of patients and controls were presented in Table 1. The case group consisted of 592 glioma patients (40.53 ± 13.90 years, 55.1% males) and 502 healthy controls (40.46 ± 18.08 years, 54.8% males). The frequency distribution of age (*p* = 0.934) and sex (*p* = 0.924) between cases and controls were no statistical differences. Among the cases, there were 378 patients with WHO 2007 grade I + II and 214 patients with grade III + IV.

**Table 1:** Characteristics of patients with glioma and health controls

Characteristics	Cases (***n*** = 592)	Controls (***n*** = 502)	***p***
**Age** (Mean ± SD, years)	40.53 ± 13.90	40.46 ± 18.08	0.934^a^
**Gender** (Males/Females)	326/266	275/227	0.924^b^
**WHO grade**			
I	43		
II	335		
III	149		
IV	65		
**Surgical method**			
STR	177		
NTR	8		
GTR	407		
**Radiotherapy**			
No	58		
Conformal radiotherapy	159		
Gamma knife	375		
**Chemotherapy**			
No	349		
Yes	243		
**Survival condition**			
Survival	41		
Lost to follow-up	24		
Death	527		

Abbreviations: *WHO* World Health Organization, *NTR* Near-total resection, *STR* Sub-total resection, *GTR* Gross-total resection

^a^ *p* values was calculated by independent samples T test

^b^ *p* values was calculated by Chi-square tests

### The genotyping results of MIR17HG variants

Five SNPs in *MIR17HG* were genotyped to determine the possible effect of *MIR17HG* variants on the risk or prognosis of glioma. The minor allele frequencies in patients and controls were displayed in Supplementary Table 2. The genotype frequencies of all the studied variants in the control group were in HWE (*p* > 0.05), and the genotyping rate exceeded 99.5%.

### The correlation between MIR17HG variants and glioma risk

The genotype and allele frequencies of these SNPs in *MIR17HG* were displayed in Table 2. Compared with the control group, the frequencies of C allele (34.9% vs 28.9%) and CC genotype (19.7% vs 9.0%) of rs7318578 were higher in glioma patients. In details, rs7318578 C allele (OR = 1.32, 95% CI: 1.10–1.58, *p* = 2.63 × 10^− 3^) and CC genotype (OR = 2.25, 95% CI: 1.54–3.31, *p* = 3.18 × 10^− 5^) were related to the increased glioma susceptibility compared with the A allele and AA genotype, respectively, and the significance still existed after the FDR controlling procedure (FDR-*p* = 0.032 and FDR-*p* = 0.001 respectively). Moreover, rs7318578 variant showed a 1.26-fold increased risk of glioma under the additive model (OR = 1.26, 95% CI: 1.07–1.49, *p* = 6.23 × 10^− 3^). There was no association between other SNPs and the risk of glioma.

**Table 2:** The effect of *MIR17HG* variants on the risk of glioma

SNP ID	Allele/Genotype	Control	Case	OR (95% CI)	***p***	FDR-***p***
rs17735387	G	829	964	1		
	A	175	220	1.08 (0.87–1.35)	0.486	0.778
	GG	341	395	1		
	GA	147	174	1.02 (0.79–1.33)	0.871	0.909
	AA	14	23	1.42 (0.72–2.80)	0.315	0.756
	GA + AA	161	197	1.06 (0.82–1.36)	0.672	0.806
	Additive	/	/	1.08 (0.87–1.34)	0.488	0.732
rs72640334	C	916	1070	1		
	A	86	110	1.10 (0.81–1.47)	0.547	0.772
	CC	418	487	1		
	CA	80	96	1.03 (0.74–1.43)	0.860	0.938
	AA	3	7	2.01 (0.51–7.83)	0.316	0.689
	CA + AA	83	103	1.07 (0.78–1.46)	0.696	0.795
	Additive	/	/	1.09 (0.82–1.47)	0.550	0.733
rs7318578	A	714	768	1		
	C	290	412	1.32 (1.10–1.58)	**2.63 × 10**^**−3**^	**0.032**
	AA	257	294	1		
	AC	200	180	0.79 (0.61–1.02)	0.073	0.438
	CC	45	116	2.25 (1.54–3.31)	**3.18 × 10**^–5*^	**0.001**
	AC + CC	245	296	1.06 (0.83–1.34)	0.654	0.826
	Additive	/	/	1.26 (1.07–1.49)	**6.23 × 10**^**−3**^	0.050
rs7336610	T	527	602	1		
	C	475	580	1.07 (0.90–1.27)	0.438	0.809
	TT	141	144	1		
	TC	245	314	1.26 (0.94–1.67)	0.119	0.476
	CC	115	133	1.13 (0.80–1.59)	0.477	0.818
	TC + CC	360	447	1.22 (0.93–1.59)	0.157	0.419
	Additive	/	/	1.07 (0.90–1.27)	0.433	0.866
rs75267932	A	879	1061	1		
	G	125	123	0.82 (0.63–1.06)	0.130	0.446
	AA	385	479	1		
	AG	109	103	0.76 (0.56–1.03)	0.073	0.438
	GG	8	10	1.01 (0.39–2.58)	0.988	0.988
	AG + GG	117	113	0.78 (0.58–1.04)	0.089	0.427
	Additive	/	/	0.82 (0.63–1.07)	0.138	0.414

Abbreviations: *SNP* Single nucleotide polymorphism, *OR* Odds ratio, *CI* Confidence interval, *FDR* False discovery

*p* values were calculated by logistic regression analysis with adjustments for age and gender

Bold *p* < 0.05 means the data is statistically significant

* After Bonferroni correction [*p* < 0.05/(5 × 4)] means the data is statistically significant

We further explored the association between glioma risk and *MIR17HG* SNPs by stratifying for age, sex and WHO grade. Among subjects of age ≥ 40 years, carriers with rs7318578 CC genotype showed a 2.46-fold increased the susceptibility to glioma compared with individuals with the AA genotype (OR = 2.46, 95% CI: 1.42–4.28, *p* = 1.41 × 10^− 3^, FDR-*p* = 0.035, Table 3). Additionally, rs17735387 was a risk factor for glioma occurrence: A vs G: OR = 1.53, 95% CI: 1.11–2.11, *p* = 9.05 × 10^− 3^; AA vs GG: OR = 3.27, 95% CI: 1.09–9.80, *p* = 0.034; GA + AA vs GG: OR = 1.57, 95% CI: 1.07–2.30, *p* = 0.021; additive: OR = 1.56, 95% CI: 1.12–2.18, *p* = 8.55 × 10^− 3^ at age <  40 years. *MIR17HG* rs7318578 C allele (OR = 1.37, 95% CI: 1.05–1.79, *p* = 0.020) and CC genotype (OR = 1.88, 95% CI: 1.08–3.28, *p* = 0.026) was associated with the increased risk of glioma in subjects aged younger 40 years. Results of multiple models showed that rs7336610 was associated with the high glioma susceptibility at age < 40 years (C vs T: OR = 1.35, 95% CI: 1.06–1.73, *p* = 0.016; TC vs TT: OR = 1.56, 95% CI: 1.02–2.39, *p* = 0.041; CC vs TT: OR = 1.72, 95% CI: 1.02–2.92, *p* = 0.044; TC + CC vs TT: OR = 1.61, 95% CI: 1.07–2.41, *p* = 0.022; additive: OR = 1.33, 95% CI: 1.02–1.73, *p* = 0.034).

**Table 3:** The effect of *MIR17HG* variants on the risk of glioma stratified by age and gender

SNP ID	Allele/Genotype	OR (95% CI)	***p***	FDR-***p***	OR (95% CI)	***p***	FDR-***p***
**Age (year)**		**≥ 40**			**< 40**		
rs17735387	G	1			1		
	A	0.79 (0.59–1.07)	0.128	0.400	**1.53 (1.11–2.11)**	**9.05 × 10**^**− 3**^	0.109
	GG	1			1		
	GA	0.73 (0.51–1.05)	0.093	0.465	1.45 (0.98–2.16)	0.065	0.142
	AA	0.87 (0.35–2.16)	0.765	0.911	**3.27 (1.09–9.80)**	**0.034**	
	GA + AA	0.74 (0.52–1.06)	0.101	0.421	**1.57 (1.07–2.30)**	**0.021**	0.101
	Additive	0.80 (0.59–1.08)	0.152	0.380	**1.56 (1.12–2.18)**	**8.55 × 10**^**−3**^	0.205
rs7318578	A	1			1		
	C	1.27 (0.99–1.62)	0.063	0.525	**1.37 (1.05–1.79)**	**0.020**	0.120
	AA	1			1		
	AC	0.64 (0.44–1.02)	0.051	0.188	0.94 (0.63–1.40)	0.754	0.952
	CC	**2.46 (1.42–4.28)**	**1.41 × 10**^–3*^	**0.035**	**1.88 (1.08–3.28)**	**0.026**	0.089
	AC + CC	0.92 (0.66–1.28)	0.606	0.947	1.15 (0.80–1.64)	0.459	0.648
	Additive	1.22 (0.97–1.54)	0.087	0.544	1.24 (0.97–1.60)	0.092	0.170
rs7336610	T	1			1		
	C	1.17 (0.93–1.48)	0.184	0.418	**1.35 (1.06–1.73)**	**0.016**	0.128
	TT	1			1		
	TC	1.35 (0.90–2.03)	0.144	0.400	**1.56 (1.02–2.39)**	**0.041**	0.109
	CC	1.35 (0.84–2.16)	0.210	0.438	**1.72 (1.02–2.92)**	**0.044**	0.106
	TC + CC	1.35 (0.92–1.98)	0.123	0.439	**1.61 (1.07–2.41)**	**0.022**	0.088
	Additive	1.16 (0.92–1.47)	0.213	0.410	**1.33 (1.02–1.73)**	**0.034**	0.102
**Gender**		**Male**			**Female**		
rs7318578	A	1			1		
	C	1.18 (0.93–1.50)	0.183	0.488	**1.53 (1.16–2.01)**	**2.49 × 10**^–3*^	**0.029**
	AA	1			1		
	AC	0.70 (0.49–1.05)	0.054	0.588	0.90 (0.61–1.33)	0.606	0.007
	CC	**1.80 (1.10–2.95)**	**0.020**	0.480	**3.08 (1.67–5.67)**	**3.19 × 10**^–4*^	0.871
	AC + CC	0.93 (0.67–1.28)	0.635	0.802	1.24 (0.87–1.77)	0.234	0.769
	Additive	1.15 (0.92–1.43)	0.226	0.493	**1.43 (1.11–1.84)**	**5.96 × 10**^**−3**^	**0.046**

Abbreviations: *SNP* Single nucleotide polymorphism, *OR* Odds ratio, *CI* Confidence interval, *FDR* False discovery

*p* values were calculated by logistic regression analysis with adjustments for age and gender

Bold *p* < 0.05 means the data is statistically significant

* After Bonferroni correction [*p* < 0.05/(5 × 4)] means the data is statistically significant

Stratified by gender (Table 3), the significant association between rs7318578 and the glioma of risk was observed in males (CC vs AA: OR = 1.80, 95% CI: 1.10–2.95, *p* = 0.020) and females (CC vs AA: OR = 3.08, 95% CI: 1.67–5.67, *p* = 3.19 × 10^− 4^, FDR-*p* = 0.046 and additive: OR = 1.43, 95% CI: 1.11–1.84, *p* = 5.96 × 10^− 3^). Especially, the association under the allele model in females was still significant (C vs A: OR = 1.53, 95% CI: 1.16–2.01, *p* = 2.49 × 10^− 3^, FDR-*p* = 0.029).

In the stratified analysis by WHO grade, rs7336610 showed a genotype difference between patients with grade III-IV and patients with grade I-II, with OR from 1.31 to 1.72 (TC vs TT: OR = 1.58, 95% CI: 1.02–2.43, *p* = 0.039; CC vs TT: OR = 1.72, 95% CI: 1.04–2.86, *p* = 0.036; TC + CC vs TT: OR = 1.62, 95% CI: 1.07–2.45, *p* = 0.022; and additive: OR = 1.31, 95% CI: 1.02–1.68, *p* = 0.035), as shown in Table 4.

**Table 4:** The effect of *MIR17HG* variants on WHO grade of glioma

SNP ID	Allele/Genotype	I-II	III-IV	OR (95% CI)	***p***	FDR-***p***
rs7336610	T	400	202	1		
	C	354	226	1.26 (1.00–1.60)	0.053	0.221
	TT	103	41	1		
	TC	194	120	**1.58 (1.02–2.43)**	**0.039**	0.244
	CC	80	53	**1.72 (1.04–2.86)**	**0.036**	0.300
	TC + CC	274	173	**1.62 (1.07–2.45)**	**0.022**	0.550
	Additive	/	/	**1.31 (1.02–1.68)**	**0.035**	0.438

Abbreviations: *SNP* Single nucleotide polymorphism, *OR* Odds ratio, *CI* Confidence interval, *FDR* False discovery

*p* values were calculated by logistic regression analysis with adjustments for age and gender

Bold *p* < 0.05 means the data is statistically significant

### The correlation between MIR17HG variants and glioma prognosis

In this study, 592 patients had complete follow-up data. The detail information for the follow-up was as following: the median, min and max follow-up time were 11 months, 2 months and 8 months, respectively. The median time to events for OS and PFS were 11 months and 8 months, respectively; total number of events for OS and DFS were 527 patients and 523 patients, respectively.

**Fig. 1:**
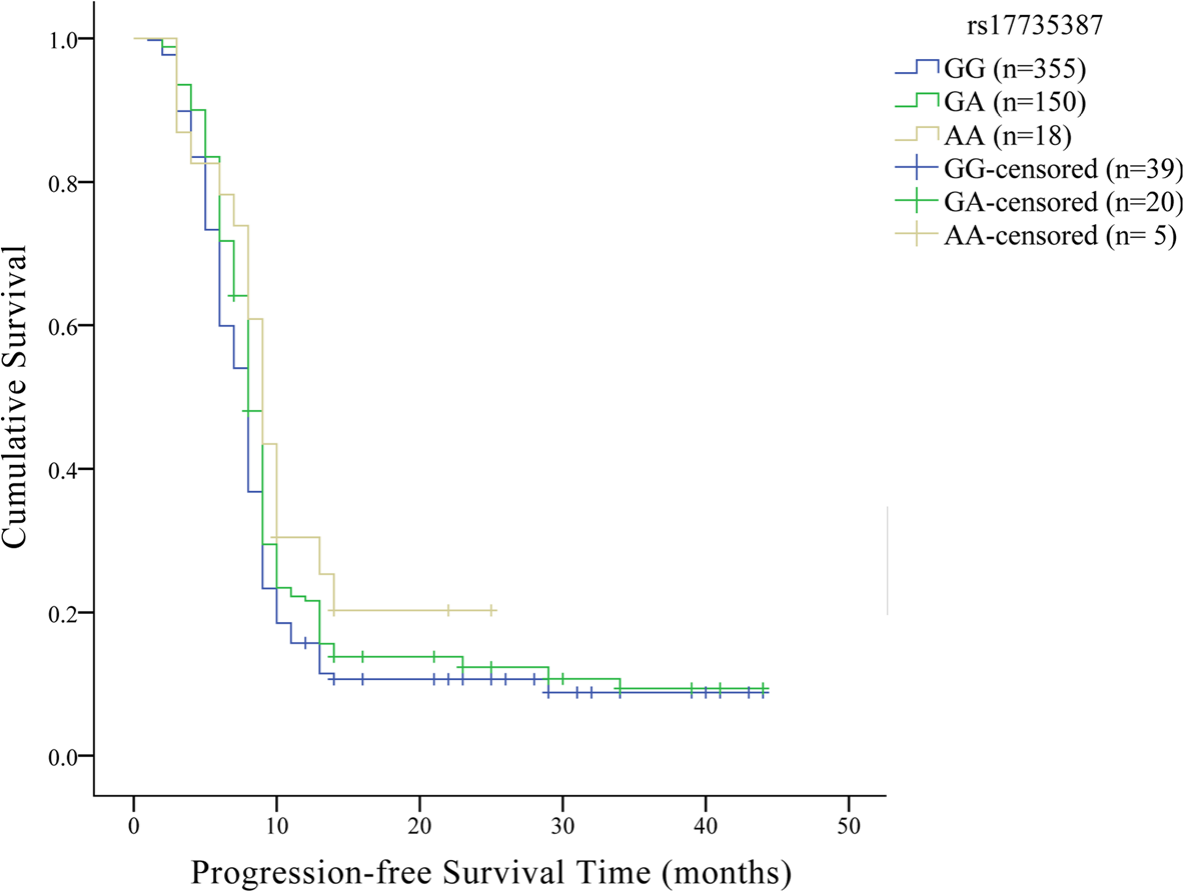
Effect of *MIR17HG* rs17735387 on the survival of overall glioma patients

Next, we investigated the correlation between *MIR17HG* variants and PFS or OS of glioma by Kaplan–Meier survival method, univariate and multivariate Cox proportional hazard model. Rs17735387 was related to the PFS of glioma (Log-rank *p* = 0.026), as shown in Fig. 1 and Table 5. Multivariate Cox proportional hazard mode adjusted for age, sex WHO grade, surgical method, use of radiotherapy and chemotherapy showed that carriers of rs17735387 GA genotype might present a longer PFS than patients with GG genotype (HR = 0.82, 95% CI: 0.68–0.99, *p* = 0.042; Table 6). No statistically significant association was found between other *MIR17HG* polymorphisms and the prognosis of glioma.

**Table 5:** Kaplan–Meier analysis of the association between *MIR17HG* variants and OS and PFS of glioma patients

SNP ID	Genotype	OS				PFS			
		Event/ Total	SR (1−/3-year)	MST (month)	Log-rank ***p***	Event/ Total	SR (1−/3-year)	MST (month)	Log-rank ***p***
**Overall**									
rs17735387	GG	356/395	0.299/0.082	11.0	0.070	355/394	0.157/0.088	8.0	**0.026**
	GA	153/174	0.360/0.101	12.0		150/170	0.216/0.094	8.0	
	AA	18/23	0.435/−	12.0		18/23	0.304/−	9.0	
rs72640334	CC	433/487	0.319/0.092	11.0	0.365	430/483	0.179/0.092	8.0	0.470
	CA	86/96	0.333/0.082	10.0		85/95	0.179/0.093	8.0	
	AA	7/7	0.143/−	10.0		7/7	0.286/−	8.0	
rs7318578	AA	263/294	0.335/0.085	12.0	0.755	262/293	0.192/0.083	8.0	0.527
	AC	160/180	0.306/0.093	11.0		159/178	0.163/0.097	8.0	
	CC	102/116	0.319/0.111	11.0		101/115	0.176/−	8.0	
rs7336610	TT	129/144	0.326/0.095	11.0	0.740	129/144	0.174/0.096	8.0	0.516
	TC	281/314	0.296/0.085	11.0		279/312	0.167/0.089	8.0	
	CC	116/133	0.381/0.095	12.0		114/130	0.221/0.098	8.0	
rs75267932	AA	425/479	0.323/0.091	11.0	0.766	422/475	0.185/0.092	8.0	0.634
	AG	92/103	0.311/0.095	10.0		91/102	0.176/0.097	8.0	
	GG	10/10	0.400/−	12.0		10/10	0.100/−	8.0	
**Low-grade glioma (I-II)**									
rs17735387	GG	232/260	0.292/0.090	11.0	**0.032**	232/260	0.158/0.093	8.0	**0.013**
	GA	86/102	0.398/0.149	12.0		84/100	0.255/0.135	9.0	
	AA	12/16	0.500/−	12.0		12/16	0.375/−	9.0	
**Females**									
rs72640334	CC	196/221	0.335/0.100	12.0	**0.035**	195/219	0.168/0.094	8.0	**0.049**
	CA	36/39	0.205/−	9.0		35/38	0.105/−	6.0	
	AA	6/6	0.167/−	10.0		6/6	−/−	8.0	
**Age ≥ 40 years**									
rs17735387	GG	217/232	0.246/0.051	10.0	**0.002**	216/231	0.134/0.059	8.0	**0.002**
	GA	78/86	0.360/0.081	12.0		78/86	0.178/0.080	8.0	
	AA	7/11	0.303/−	16.0		7/11	0.545	13.0	

Abbreviations: *OS* Overall survival, *PFS* Progression free survival, *SR* Survival rate, *MST* Median survival time

Log-rank *p* values were calculated using the Chi-Square test

Bold *p* < 0.05 indicates statistical significance

**Table 6:** Cox proportional hazards model of the association between *MIR17HG* variants and OS and PFS of glioma patients

SNP ID	Genotype	Univariate				Multivariate ^**a**^			
		OS		PFS		OS		PFS	
		HR (95% CI)	***p***	HR (95% CI)	***p***	HR (95% CI)	***p***	HR (95% CI)	***p***
**Overall**									
rs17735387	GG	1		1		1		1	
	GA	0.85 (0.70–1.03)	0.097	0.83 (0.69–1.01)	0.059	0.84 (0.69–1.01)	0.067	**0.82 (0.68–0.99)**	**0.042**
	AA	0.70 (0.43–1.12)	0.136	0.66 (0.41–1.07)	0.089	0.84 (0.46–1.19)	0.211	0.71 (0.44–1.14)	0.158
rs72640334	CC	1		1		1		1	
	CA	1.08 (0.86–1.36)	0.508	1.07 (0.85–1.35)	0.560	1.08 (0.85–1.37)	0.520	1.09 (0.86–1.38)	0.467
	AA	1.56 (0.74–3.29)	0.247	1.44 (0.68–3.05)	0.335	1.25 (0.58–2.66)	0.569	1.20 (0.56–2.56)	0.633
rs7318578	AA	1		1		1		1	
	AC	1.07 (0.88–1.30)	0.493	1.11 (0.91–1.35)	0.310	1.07 (0.88–1.30)	0.516	1.10 (0.90–1.34)	0.353
	CC	1.03 (0.82–1.30)	0.776	1.04 (0.82–1.30)	0.762	1.05 (0.83–1.32)	0.701	1.04 (0.83–1.31)	0.725
rs7336610	TT	1		1		1		1	
	TC	1.00 (0.81–0.23)	0.98	0.99 (0.81–1.23)	0.957	0.96 (0.78–1.18)	0.703	0.96 (0.78–1.18)	0.698
	CC	0.93 (0.72–1.19)	0.549	0.89 (0.69–1.15)	0.381	0.91 (0.71–1.17)	0.480	0.89 (0.69–1.15)	0.375
rs75267932	AA	1		1		1		1	
	AG	1.07 (0.85–1.33)	0.585	1.07 (0.85–1.34)	0.568	1.04 (0.83–1.31)	0.727	1.05 (0.84–1.32)	0.671
	GG	1.14 (0.61–2.14)	0.675	1.24 (0.66–2.32)	0.502	1.17 (0.62–2.20)	0.633	1.24 (0.66–2.34)	0.502
**Low-grade glioma (I-II)**									
rs17735387	GG	1		1		1		1	
	GA	**0.77 (0.60–0.99)**	**0.042**	**0.75 (0.58–0.97)**	**0.024**	**0.75 (0.58–0.96)**	**0.024**	**0.73 (0.57–0.94)**	**0.016**
	AA	0.64 (0.36–1.15)	0.138	0.62 (0.35–1.11)	0.110	0.68 (0.38–1.22)	0.195	0.70 (0.39–1.26)	0.233
**Females**									
rs72640334	CC	1		1		1			
	CA	1.49 (1.05–2.14)	**0.027**	1.48 (1.03–2.12)	**0.034**	0.89 (0.65–1.21)	0.454	0.88 (0.65–1.20)	0.427
	AA	1.50 (0.66–3.38)	0.332	1.35 (0.60–3.05)	0.470	2.05 (0.28–4.87)	0.477	2.62 (0.36–8.99)	0.342
**Age ≥ 40 years**									
rs17735387	GG	1		1		1		1	
	GA	1.30 (1.00–1.68)	0.500	0.80 (0.62–1.04)	0.098	0.77 (0.59–1.00)	**0.047**	0.79 (0.61–1.03)	0.084
	AA	1.00 (0.74–1.35)	0.993	0.36 (0.17–0.76)	**0.007**	0.46 (0.22–1.00)	**0.049**	0.45 (0.21–0.97)	**0.042**

Abbreviations: *OS* Overall survival, *PFS* Progression free survival, *HR* Hazard ratio, *CI* Confidence interval

^*a*^ *p* values were calculated by Cox multivariate analysis with adjustments for gender, age, WHO grade, surgical method, use of radiotherapy and chemotherapy

Bold *p* < 0.05 indicates statistical significance

**Fig. 2:**
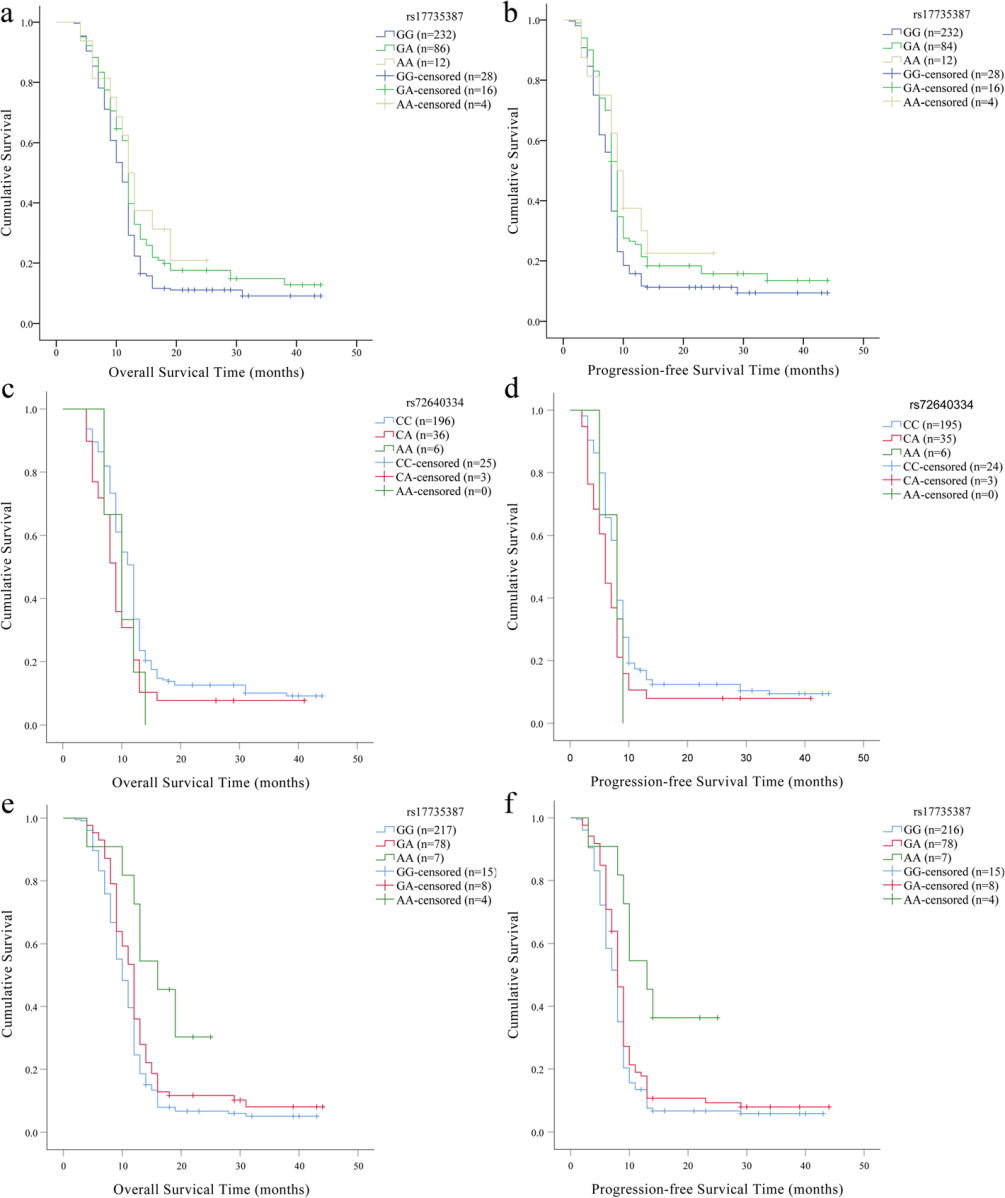
Stratified by age, sex and grade, effect of *MIR17HG* rs17735387 on the survival of patients. The survival curve of overall survival for patients with I-II glioma (**a**), female patients (**c**), patients with age ≥ 40 years (**e**) and of progression free survival for patients with I-II glioma (**b**), female patients (**d**), patients with age ≥ 40 years (**f**)

In patients with low-grade glioma (I-II), the Kaplan–Meier method (Table 5) revealed the association between *MIR17HG* rs17735387 and OS (Log-rank *p* = 0.032, Fig. 2a) or PFS (Log-rank *p* = 0.013, Fig. 2b). Univariate Cox proportional hazard model presented that the GA genotype of rs17735387 might had a better OS (HR = 0.77, *p* = 0.042) and PFS (HR = 0.75, *p* = 0.024) when compared with GG genotype among patients with I-II glioma (Table 6). Moreover, the multivariate Cox proportional hazard model also displayed that a better prognosis for glioma was also seen for rs17735387-GA genotype (OS: HR = 0.75, *p* = 0.024 and PFS: HR = 0.73, *p* = 0.016). However, no association between *MIR17HG* polymorphisms and the prognosis of glioma in high-grade glioma patients was found.

The age and sex stratified analyses were performed to assess the association between *MIR17HG* polymorphisms and the prognosis of glioma. In female patients, Kaplan–Meier method (Table 5) revealed the association of rs72640334 with OS (Log-rank *p* = 0.035, Fig. 2c) or PFS (Log-rank *p* = 0.049, Fig. 2d). The results of univariate Cox proportional hazard model showed that rs72640334 was related to the poor prognosis (OS, HR = 1.49, *p* = 0.027 and PFS, HR = 1.48, *p* = 0.034, Table 6). Kaplan–Meier method (Table 5) revealed the association between rs17735387 and OS (Log-rank *p* = 0.002, Fig. 2e) or PFS (Log-rank *p* = 0.002, Fig. 2f) among patients with age ≥ 40 years. In the subgroup of patients with age ≥ 40 years, GA genotype (multivariate: OS, HR = 0.77, *p* = 0.047) and AA (univariate: PFS, HR = 0.036, *p* = 0.007; multivariate: OS, HR = 0.46, *p* = 0.049 and PFS, HR = 0.45, *p* = 0.042, Table 6) genotype of rs17735387 were associated with a better prognosis.

## Discussion

This study explored the possible relationship between *MIR17HG* variants and the occurrence and prognosis of glioma in a Chinese Han population. Our data revealed that rs7318578, rs17735387 and rs7336610 polymorphisms were associated with the increased susceptibility to glioma. We also found that rs17735387 was related to a better prognosis of patients with glioma. To our knowledge, we firstly reported that *MIR17HG* polymorphisms might be related to glioma susceptibility and patients’ survival.

*MIR17HG* gene is also called *c13orf25* and *Oncomir-1*, which encodes a polycistronic miR-17-92 cluster encompassed six miRNAs (miR-17, miR-18a, miR-19a, miR-20a, miR-19b-1, and miR-92a-1). The miR-17-92 cluster was deregulated in glioma, indicating that these miRNA played a key role of in gliomagenesis [19, 20]. Schulte JH et al. reported that miR-17-92 cluster amplification in neuroblastomas was associated with a poor prognosis [21]. lncRNA MIR17HG was upregulated in glioma tissues and cell lines, and acted as competing endogenous RNA (ceRNA) to sponge miR-346/miR-425-5p in regulating the malignant of glioma [14]. Yuze Cao et al. reported that lncRNA MIR17HG-mediated ceRNA network was identified as a potential prognostic biomarker for glioblastoma [22]. Moreover, Xue Leng et al. observed that MIR17HG was highly expressed in glioma and participated in piR-DQ590027/ lncRNA MIR17HG/miR-153(miR-377)/FOXR2 pathway which involved in regulating the permeability of glioma-conditioned normal blood-brain barrier [23]. These results suggested that lncRNA MIR17HG could be of pathogenic importance in the development and prognosis of glioma. Several previous studies have reported the effect of *MIR17HG* genetic polymorphisms on the risk of various disease including tumors [24, 25], but not in glioma.

Considering the importance of *MIR17HG* in the carcinogenic process of glioma, we hypothesized that *MIR17HG* polymorphisms might also are associated with glioma development. Here, we explored the relationship between five SNPs in *MIR17HG* and the risk and prognosis of glioma in a Chinese Han population. We found that rs7318578 might had a higher susceptibility to glioma. The incidence rates of glioma, that is, the rate of newly diagnosed tumor, are associated with increasing age and male gender [26]. We further analyzed whether the genotypic effects of *MIR17HG* on the risk of glioma were dependent on age and sex. We found that rs7318578 was related to the increase risk of glioma in the subjects with age ≥ 40 years or in females. In addition, rs17735387 and rs7336610 also had a higher susceptibility to glioma in the subgroup aged < 40 years. These indicated that the effect of *MIR17HG* polymorphisms on glioma occurrence might present age and sex difference. More importantly, we found that rs17735387 was related to the better prognosis of patients with glioma, particularly in low-grade glioma. Previously, rs7336610 was reported to be associated with the risk of multiple myeloma and breast cancer, while rs17735387 had no relationship with the risk and prognosis of multiple myeloma [16, 24]. These results suggested that *MIR17HG* polymorphisms might have a different effect on the occurrence of different cancer types. However, our findings need further studies to confirm.

Inevitably, some limitations should not be ignored. First, all individuals including glioma patients and healthy controls were from the same hospital, therefore the selection bias cannot be ruled out. Second, due to the lack of data on environmental exposure and diet, the interaction between environment and genetics needs to be further explored in larger prospective studies. Third, the effect of these SNPs on miR-17-92 cluster or lncRNA MIR17HG was not assessed.

## Conclusion

In conclusion, we reported that *MIR17HG* rs7318578 might be a risk factor for the susceptibility of glioma and rs17735387 was associated with the longer survival of glioma among Chinese Han population. Our study firstly provided evidence about the effect of *MIR17HG* polymorphisms on the risk and prognosis of glioma, which might help to enhance the understanding of *MIR17HG* gene in gliomagenesis. In subsequent studies, we will continue to collect samples and follow up to further validate our findings and further explore the function of these *MIR17HG* SNPs in glioma in a larger sample size.

## Supplementary information

**Additional file 1: Table S1.** Primers sequence for PCR amplification and extension of *MIR17HG* variants. **Table S2.** The details of candidate SNPs in the *MIR17HG* gene.

## Data Availability

All the data regarding the findings are available within the manuscript. Anyone who is interested in the information should contact the corresponding author.

## Abbreviations

SNP: Single-nucleotide polymorphisms
OR: Odds ratio
CI: Confidence intervals
HR: Hazard ratios
OS: Overall survival
PFS: Progression-free survival
MAFs: Minor allele frequencies
HWE: Hardy–weinberg equilibrium
