# Supplementary material for: Genetic variants in MIR17HG affect the susceptibility and prognosis of glioma in a Chinese Han population
Source: BMC Cancer. 2020 Oct 9;20:976. doi: 10.1186/s12885-020-07417-9 (PMC7547478; doi:10.1186/s12885-020-07417-9)
Supplement: Supplementary file 1 — Additional file 1: Table S1. Primers sequence for PCR amplification and extension of MIR17HG variants. Table S2. The details of candidate SNPs in the MIR17HG gene. [file 12885_2020_7417_MOESM1_ESM.docx]

Supplementary **Table 1. Primers sequence for PCR amplification and extension of *MIR17HG* variants**

| SNP ID | First Primer(5'-3') | Second Primer (5'-3') | UEP_DIR | UEP SEQ (5'-3') |
| --- | --- | --- | --- | --- |
| rs17735387 | ACGTTGGATGGCTTTCTTTCCAAATATAGGC | ACGTTGGATGAGCCTTAACTATTTGGAGGG | R | AATAGAAAGTTGTACATGCAAA |
| rs72640334 | ACGTTGGATGGCTTAAGAACTCTGCTAATG | ACGTTGGATGCTATCATTCTGGAGTTGATG | R | CACTGTTCATTTCACATCCT |
| rs7318578 | ACGTTGGATGGAAATCATCCAGCAGGCTTC | ACGTTGGATGCAGCATGGTCTGGTAGTTTG | R | TCCTATCACACTGTTCCA |
| rs7336610 | ACGTTGGATGACAGCGTTTCACCATGTCGG | ACGTTGGATGAAAAAGTTCCGGCTGGACAC | R | CTCCTGACCTCAGGTAATCC |
| rs75267932 | ACGTTGGATGTTAGAGAGAATGCCGCTCTG | ACGTTGGATGCCCAACCCTAAATTCCATGC | F | cccCCGCTCTGTTTAAAGCAATGTGTA |

SNP: single nucleotide polymorphism; UEP: unextended mini sequencing primer; DIR: direction; SEQ, sequence.

Supplementary **Table 2. The details of candidate SNPs in the *MIR17HG* gene**

| SNP ID | Chr: Position | Alleles  (minor/major) | MAF | | Call rate | HWE | | |
| --- | --- | --- | --- | --- | --- | --- | --- | --- |
|  |  |  | Cases | Controls |  | O(HET) | E(HET) | *p* |
| rs17735387 | 13:91353800 | A/G | 0.186 | 0.174 | 100.0% | 0.293 | 0.288 | 0.877 |
| rs72640334 | 13:91352674 | A/C | 0.093 | 0.086 | 99.7% | 0.160 | 0.157 | 1.000 |
| rs7318578 | 13:91353215 | C/A | 0.349 | 0.289 | 99.8% | 0.398 | 0.411 | 0.515 |
| rs7336610 | 13:91352883 | C/T | 0.491 | 0.474 | 99.8% | 0.489 | 0.499 | 0.655 |
| rs75267932 | 13:91351812 | G/A | 0.104 | 0.125 | 100.0% | 0.217 | 0.218 | 0.840 |

SNP, Single nucleotide polymorphism; MAF, Minor allele frequency; HWE, Hardy-Weinberg equilibrium.
